# microRNAs in cardiac regeneration and cardiovascular disease

**DOI:** 10.1007/s11427-013-4534-9

**Published:** 2013-08-22

**Authors:** GengZe Wu, Zhan-Peng Huang, Da-Zhi Wang

**Affiliations:** 1 Department of Cardiology, Boston Children’s Hospital, Harvard Medical School, Boston, MA 02115, USA; 2 Department of Cardiology, Chongqing Institute of Cardiology, Daping Hospital, Third Military Medical University, Chongqing 400042, China; 3 Harvard Stem Cell Institute, Harvard University, Cambridge, MA 02138, USA

**Keywords:** microRNA, cardiac regeneration, cardiac remodeling, cardiovascular disease

## Abstract

microRNAs (miRNAs) are a class of small non-coding RNAs, which have been shown important to a wide range of biological process by post-transcriptionally regulating the expression of protein-coding genes. miRNAs have been demonstrated essential to normal cardiac development and function. Recently, numerous studies indicate miRNAs are involved in cardiac regeneration and cardiac disease, including cardiac hypertrophy, myocardial infarction and cardiac arrhythmia. These observations suggest miRNAs play important roles in cardiology. In this review, we summarize the recent progress of studying miRNAs in cardiac regeneration and cardiac disease. We also discuss the diagnostic and therapeutic potential of miRNAs in heart disease.

Historically, we have been focusing on protein-coding genes over the past decades and proteins have often been viewed as the final gene products that mediate most of the biological function in a given cell. However, more and more recent studies indicated that non-coding RNAs (ncRNAs) participate in the regulation of the expression of protein- coding genes therefore play essential roles in many biological processes. These findings uncovered that noncoding RNAs orchestrate a hidden layer of gene regulation network [1,2]. microRNAs (miRNAs), a class of small non-coding RNAs (~20 nucleotides) [3,4], were first discovered in *C. elegans* two decades ago [5,6]. Now we know that miRNAs are present in virtually all plants [7] and animals [8–10]. Many miRNAs are evolutionary conserved, indicating they may serve as an ancient component of genetic regulation [11–14]. miRNAs are transcribed by RNA polymerase II (RNA-pol II) as primary transcripts (primiRNAs) [15], which contain stem-loop hairpin structure, and then subsequently processed by RNase III enzymes Drosha and Dicer [16–19]. The processed products are ~22 nt long miRNA duplexes with 2 nt overhangs on the 3′ end [20,21]. Dicer also contributes to the loading of mature miRNAs into the RNA-induced silencing complex (RISC). RISC is responsible for the gene silencing observed due to miRNA expression and RNA interference [22,23]. After loading into the RISC complex, miRNAs guide the RISC complex to their target genes by binding to imperfect complementary sites within the 3′ untranslated regions (3′UTRs) [24]. Studies have shown that miRNA can also bind to the 5′ untranslated regions (5′UTRs) and even the open reading frames (ORFs) of their target mRNAs [25]. miRNAs repress the expression of their target genes by mRNA destabilization and/or translational repression [24,26]. Moreover, miRNAs were also reported to cause histone modification and/or DNA methylation of promoter sites, which affects the expression of target genes [27,28].

## 1 miRNAs in cardiac development

Global disruption of the expression of all miRNAs in the heart is the first step to understand the function of miRNAs in cardiac development and physiology. Dicer, an RNase III endoribonuclease is a critical enzyme for the maturation of most miRNAs. Conventional deletion of Dicer caused early embryonic lethality in mice [29] and zebrafish [30,31], prior to the development of the heart. In order to study the function of miRNAs in cardiac development, studies using cardiac- specific promoter-driven Cre line, such as Nkx2.5-Cre and α-MHC-Cre, to knockout Dicer in cardiac linage have been performed. Disrupting miRNA expression in early embryonic stage using Nkx2.5-Cre leads to improperly compacted ventricular myocardium in mutant embryos [32]. While α-MHC-Cre-mediated conditional deletion of Dicer causes postnatal lethality due to dilated cardiomyopathy and heart failure [33]. Additionally, tamoxifen-induced cardiacspecific deletion of Dicer in adult hearts induces spontaneously cardiac hypertrophy and fetal gene expression [34]. Moreover, cardiac-specific knockout of Drosha leads to similar cardiac defects with that of cardiac-specific Dicer mutant, further supporting the importance of miRNAs in cardiac development and function [35].

Studies of miRNA expression profiling have indicated that although many miRNAs are expressed ubiquitously throughout the mammalian organisms, some miRNAs have tissue-specific expression patterns [36]. Focusing on striated muscles like cardiomyocytes and skeletal muscle cells, previous studies found that miR-1/133 cluster is the most abundantly expressed miRNA in the heart whose expression was detected as early as embryonic day 13.5 (E13.5) [37]. The expression of this miRNA cluster can be regulated by multiple myogenic transcription factors, including MyoD [38], Mef2 [39], and SRF [40]. miR-1, miR-133, together with miR-206 [41], miR-208a/b [42,43] and miR-499 [44] have been identified specifically expressed in striated muscles (and therefore referred to as “myomiRs”). Gain- and loss-of-function studies demonstrated that myomiRs play key roles in cardiomyocyte proliferation, cardiac morphogenesis and stress responsive cardiac remodeling [40,42,43]. Interestingly, recent reports showed that miRNAs, which are not restricted to striated muscles, are also required for the normal cardiogenesis. miR-138 is required for the elongation of ventricular cardiomyocytes by repressing the expression of cspg2 and notch1b, which are normally restricted to the atrioventricular canal region, in the ventricle in zebrafish [45]. Loss-of-function of miR-218 in zebrafish affects the endocardial migration, and therefore affects the heart tube formation by regulating the Vegf singling [46].

## 2 miRNAs mediate cardiac regeneration

Mammalian adult cardiomyocytes are terminally differentiated cells with very limited regenerative ability. In response to injury and cell loss, adult mammalian hearts are unable to fully regenerate. However, recent evidences indicate that human cardiomyocytes are able to proliferate during the postnatal life [47,48]. Intriguingly, it was discovered that in rodent models hearts before postnatal day 7 have a good regenerative ability. Young hearts can regenerate the cardiac tissue without the formation of scar (fibrosis) after resection of the ventricle apex. However, the cardiac regenerative ability is lost by 7 d of age due to a significant decrease of the proliferative rate of cardiomyocytes [49].

Both protein-coding genes and non-coding RNA genes have been suggested to participate in the regulation of cardiac regeneration. For protein-coding genes, inhibition of p38 MAP kinase was found to induce mitosis in adult mammalian cardiomyocytes [50]. Treatment with FGF1 and p38 MAP kinase inhibitors regenerates the heart, reduces scarring, and improves cardiac function in rats with cardiac injury [51]. Non-coding RNAs, especially miRNAs, have also been found playing an important role in cardiac regeneration. miRNA profiling analyses indicated that the expression of miRNAs is regulated during cell cycle exit of cardiomyocyte shortly after born. Through profiling and comparing miRNA expression between postnatal day 1 and day 10 rat cardiomyocytes, Porrello et al. [52] identified members of the miR-15 family, including miR-195, miR-15a, miR-15b, miR-16 and miR-497, as important regulators of postnatal cardiomyocyte mitotic arrest. Further gain- and loss-of-function studies demonstrated that cardiomyocytes proliferation can be inhibited by this family of miRNAs through the repression of multiple cell cycle regulators, including the checkpoint kinase 1 (Chek1). Interestingly, miR-15 family was also shown up-regulated in cardiac ischemia and heart failure [53] and it was reported that miR-15 induces apoptosis by targeting anti-apoptotic factor Bcl2 [54]. Together, these studies suggest that the miR-15 family may play distinct roles in cardiomyocytes at different developmental and/or pathological conditions.

To further investigate if miRNAs can increase cardiomyocytes proliferation, Eulalio et al. [55] performed a high-content, fluorescence-microscopy-based, high-throughput screening in neonatal rat cardiomyocytes using a whole-genome miRNA library. They identified about 40 miRNAs that strongly increased both DNA synthesis and cytokinesis in neonatal mouse and rat cardiomyocytes [55]. Two of these miRNAs, miR-590 and miR-199a, were further demonstrated to induce cardiomyocyte proliferation both *in vitro* and *in vivo*. Most recently, our group also discovered that other miRNAs can increase cardiomyocytes proliferation. Using both transgenic (TG) and knockout (KO) mice model, we demonstrated that miR-17-92 cluster is required for and sufficient to induce cardiomyocyte proliferation. More specifically, we identified miR-19a/b as the major contributors among the miR-17-92 cluster to the regulation of the cardiomyocyte proliferation. We further identified the tumor suppressor PTEN as a direct target of miR-17-92 repressed. These studies uncovered that miR-17-92 plays a key role in the regulation of cardiomyocytes proliferation in embryonic, postnatal and adult hearts [56].

Over the past several years, the approaches to differentiating embryonic stem cells and induced pluripotent stem cells (iPSCs) into cardiomyocytes to compensate the loss of cardiomyocytes during cardiac injury have raised the hope for patients with cardiac infarction and heart failure [57,58]. However, the potential tumorigenicity is a big concern for these strategies of cardiac regeneration [59,60]. Most recently, an exciting breakthrough was achieved in which Ieda et al. [61] found a new strategy to direct reprogram fibroblast (CF) into cardiomyocyte (CM) through the combination of three cardiac-specific transcriptional factors, Gata4, Mef2c and Tbx5 (GMT) *in vitro*. Intriguingly, Qian et al. [62] and Song et al. [63] demonstrated that the direct reprogramming was also achievable *in vivo*. These investigators reported that they were able to use three factors (GMT) or four cardiac transcription factors, GATA4, HAND2, MEF2C, and TBX5 (GHMT) respectively, to reprogram cardiac fibroblasts or even mouse tail-tip fibroblasts into beating cardiomyocyte-like cells *in vivo*. More importantly, reprogramming cardiac fibroblasts into cardiomyocytes *in vivo* was shown to improve cardiac function and reduce cardiac fibrosis in a mouse model of myocardial infraction. It is not known whether miRNAs are involved in the process of reprogramming which is induced by cardiac transcription factors.

Given their characteristics of tiny and mighty, it is not surprising that a recent report indicated that a combination of miR-1, miR-133, miR-208, and miR-499 was able to directly induce the cellular reprogramming of fibroblasts into cardiomyocyte-like cells *in vitro* [64]. The investigators demonstrated that miR-1 alone is sufficient to induce the fibroblast to cardiomyocyte reprogramming. However, this reprogramming efficiency was dramatically enhanced when miRNAs 133, 208 and 499 were added. Interestingly, the process of reprogramming was further enhanced about 10-fold after JAK inhibitor I treatment. Moreover, administration of miRNAs into ischemic mouse myocardium resulted in direct conversion of cardiac fibroblasts to cardiomyocytes *in situ*. Recently, Nam et al. [65] used a combination of transcription factors and miRNAs to induce the direct reprogramming of fibroblasts into cardiomyocyte-like cells. They treated human fibroblasts with four transcriptional factors, GATA-4, Hand2, Tbx5 and Myocardin [66], together with two miRNAs, miR-1 and miR-133. Portion of the treated human fibroblasts were reprogrammed into cells with sarcomere-like structures, showing spontaneous contractility after 4–11 weeks in culture. Besides the phenotypic changes, the investigators found that the transcriptome of reprogrammed cells also shifted toward cardiomyocytes. These data indicated miRNAs could function with cardiac transcriptional factors to synergistically control cardiac reprogramming.

Unlike adult skeletal muscle, which contains a large population of satellite cells to serve as stem cells to regenerate the lost muscle mass in response to injury, the adult heart only has a very limited population of stem cells or progenitor cells. Studies indicated that these cells can be differentiated into cardiomyocyte *in vitro*. Interestingly, miRNAs are shown to contribute to the differentiation of cardiac stem cells (CSCs) into mature, working cardiomyocytes. Hosoda et al. [67] identified that miR-499 promotes the differentiation of human CSCs (hCSCs) into mechanically integrated cardiomyocytes by repressing its targets, Sox6 and Rod1. More importantly, the miR-499-overexpressed hCSCs injected into the infracted mouse hearts could enhance cardiac differentiation and further contribute to a better improvement of the cardiac function of the injured hearts.

## 3 miRNAs regulate cardiac remodeling

Cardiac remodeling, which is defined as alteration in the structure (dimensions, mass, shape) of the heart, is one of the major responses of the heart to biomechanical stress and pathological stimuli. Among them, cardiac hypertrophy is anatomically defined as an increase in the thickness of the cardiac ventricular wall, owing to enlargement of myocyte size and/or increased fibrosis. Sustained cardiac hypertrophy often leads to end stage heart failure. To investigate the involvement of miRNAs in this process, genome-wide profiling of miRNA expression has been performed and dysregulated miRNAs were identified during cardiac remodeling [68,69]. For instance, miR-21, a miRNA upregulated during hypertrophy, was shown to promote cardiac fibroblast survival through enhancing the ERK-MAP kinase activity [70]. Interestingly, inhibition of miR-21 via an antagomir was shown to repress cardiac hypertrophy and fibrosis *in vivo* in response to stress, however, these results could not be verified through genetic deletion of miR-21 in mice [71], indicating that miR-21 may not be essential for the pathological remodeling of the heart. Another study showed that isoproterenol-induced cardiac hypertrophy could be repressed when miR-23a was knocked down. miR-23a represents another miRNA up-regulated during hypertrophy and the repressive effect of miR-23a in cardiac hypertrophy was suggested, at least in part, due to the repression of MuRF1, an anti-hypertrophic factor [72]. Moreover, cardiac-specific overexpression of miR-195, another miRNA induced in hypertrophic hearts, in transgenic mice model induces significantly cardiac hypertrophy and dilated cardiomyopathy with unknown mechanism [53]. Recently, we and others [73,74] demonstrated that miR-22, a miRNA enriched in cardiomyocytes but only mildly up-regulated during cardiac hypertrophy, significantly promotes cardiac hypertrophy *in vitro*. Consistently, cardiac-specific knockout of miR-22 in mice showed the repressed cardiac hypertrophy was accompanied with accelerated dilation [73]. Conversely, cardiac-specific overexpression of this miRNA induced spontaneous hypertrophy growth in the heart [75]. Additional studies showed that miR-22 represses a broad spectrum of target genes, including Sirt1, HDAC4, PPARa and Purb, a negative regulator of SRF [73,75].

Conversely, unlike those up-regulated miRNAs, the expression of miR-93, miR-181 and muscle-specific micro- RNAs miR-1 and miR-133a is down-regulated during cardiac hypertrophy [53,76,77]. The miR-133a is coded by two genomic loci, miR-133a-1 and miR-133a-2. Studies showed that inhibition of miR-133a leads to hypertrophy both *in vitro* and *in vivo*, probably through the derepression of RhoA, Cdc42, and NELFA/WHsc2 [76]. Genetic studies indicated that deletion of both miR-133a-1 and miR-133a-2, but not each single one alone, leads to phenotypically abnormal, even embryonic lethal in mice [78]. All these data suggest that miR-133a is not only necessary for cardiac development, but also required for normal cardiac functional maintenance in adult hearts. miR-208a, whose expression was not altered in cardiac hypertrophy, has been demonstrated to be involved in stress-dependent cardiac hypertrophy by targeting thyroid hormone receptor associated protein 1 (THRAP1). Both gain- and loss-of-function studies demonstrated that miR-208 is not only sufficient to induce cardiac hypertrophy but also required for the hypertrophic growth under stress [42,43].

Cardiac fibrosis, which is defined as abnormal deposition of collagen by cardiac fibroblast, is often observed to replace the “drop-out” of cardiomyocytes during cardiac remodeling. Many genes have been reported to participate in the regulation of this process. It is not surprising that miRNAs were reported to regulate cardiac fibrosis in recent years. Connective tissue growth factor (CTGF) is a key molecule in the process of fibrosis and therefore seemingly serves as an attractive therapeutic target [79–81]. However, it was unknown how CTGF transcripts were regulated posttranscriptionally. Duisters et al. [82] showed that miR-133 and miR-30 were involved in myocardial matrix remodeling through regulating CTGF. Both miR-133 and miR-30 were found consistently down-regulated in several models of heart failure and pathological hypertrophy. Knockdown of these miRNAs resulted in a strong increase of CTGF levels. Conversely, overexpression of miR-133 and miR-30c repressed the production of collagens, which was accompanied with a decrease in CTGF expression levels. Moreover, the miR-29 family, which was predominantly expressed in cardiac fibroblasts [83], is significantly down-regulated in the fibrotic border zone of infracted hearts. Intriguingly, many of the miR-29 downstream target genes, such as FBN1, COL1A1, COL1A2, ELN and COL3A1, are upregulated after myocardial infarction, suggesting that they may contribute to miR-29-mediated cardiac fibrosis. Taken together, these studies indicate that miRNAs are not only important for cardiovascular development, but also essential factors for cardiac remodeling.

## 4 miRNAs regulate ischemia and conduction system

Ischemia is an independent risk factor of cardiovascular events, which leads to myocardial infarction (MI) and ischemia- reperfusion (I/R) injury. Several miRNAs are reported to participate in the regulation of these pathologic processes, especially the cardiomyocyte apoptosis following MI and I/R injury. miR-92a, a member of miR-17-92 cluster involved in cardiomyocyte proliferation, also participated in the control of cardiomyocyte survival through targeting integrin subunit α5 and eNOS [84]. Inhibition of miR-92a by antagomir has improved cardiac function and deduced cardiomyocyte apoptosis after myocardial infraction in mice. miR-21 serves as an anti-apoptotic factor in MI animal models through targeting PDCD4 and repressing its expression [85]. Unlike miR-21, miR-1/206 acts as a pro-apoptotic factor by repressing the anti-apoptosis gene *IGF-1* in infracted heart. Both IGF-1 loss-of-function and miR-1/206 gain-of-function can increase caspase-3-mediated apoptotic signaling pathway [86]. Additionally, miR-320 is downregulated after I/R injury. Gain- and loss-of-function studies demonstrated that miR-320 promotes cardiomyocyte apoptosis via maintaining the HSP20 levels [87].

The cardiac conduction system can be damaged following cardiac injury, such as cardiac ischemia or acute MI. Cellular necrosis in the lesion region is able to cause the dysfunction of the whole cardiac conduction system, including sinoatrial node, atrioventricular node and His-Purkinje system. Some of the defects include that the electrical signals could not be conducted smoothly through this conduction system, following a series of arrhythmia. miRNAs have been shown to participate in this process and the proper expression of miRNAs is critical for sustaining the normal function of cardiac conduction system. For instance, it has been reported that miR-1 and miR-133 target several ion channel and gap-junction associated genes, such as HCN2, HCN4, KCNJ2, ERG and GJA1 (Cx43) [88–90]. Overexpression of miR-1 in infracted myocardium can promote arrhythmogenesis, whereas arrhythmia could be alleviated through deleting endogenous miR-1 [90]. miR-208a, another cardiac-specific miRNA, has also been demonstrated to play an important role in arrhythmogenesis [42], especially in the process of atrial depolarization, by regulating the expression of Connexin-40 (GJA5). Therefore, studies have established the role of miRNAs in the development and maintenance of the cardiac conduction system as well as in the pathology of cardiac ischemia.

## 5 Conclusion and perspectives

miRNA research has rapidly emerged as an important area within the field of non-coding RNAs. These newly characterized “small” regulators have proven to play “big” roles in multiple levels of cellular biological behavior, from cell proliferation, differentiation and apoptosis, to the maintenance of stem cell self-renewal. From cardiac point of view, miRNAs have been found to participate in nearly all processes of cardiovascular biology, such as heart development, cardiomyocyte regeneration, cardiac remodeling, as well as stress induced injury and heart diseases. However, the identified miRNAs and miRNA-targets thus far are just the tip of the iceberg, and numerous questions concerning the relationship between miRNAs and cardiac function remain to be answered.

miRNAs could be utilized as bio-markers for the diagnosis of cardiovascular disease, given that the expression of many miRNAs is altered in varieties of cardiac biologic processes and disease conditions. The “circulating mi- RNAs” are stable in mammalian serum and plasma, which are much easier to acquire, raising the possibility that they could serve as biomarkers for heart disease diagnosis and prediction [91,92]. Recently, a clinical study has already made progress toward this possibility by showing the circulating miR-192 level is correlated with the development of ischemic heart failure after acute myocardial infarction in human patients [93]. The research for translational medicine of miRNAs has emerged rapidly in recent years. Though the therapeutic potential of miRNAs in cardiovascular disease remains debatable, much remarkable progress has been made. Cardiologists are now attempting to use miRNAs and/or their inhibitors to treat heart diseases. Along with that, many efficient techniques to manipulate miRNA levels *in vivo* have been developed. Among them, antagomirs, which knock down targeted miRNAs via sequestering them from the functional complex, were shown to be stable in the blood [94]. Conversely, miRNA mimic, a synthesized chemically modified double-stranded oligonucleotide, can achieve the gain-of-function of specific miRNA *in vitro* and *in vivo* [95,96]. Additionally, rAAV9 vector has been demonstrated of high affinity for myocardium, which means it can be used for delivering miRNA-related therapeutic molecules to the heart specifically through intravenous injection [97]. With these efficient strategies for gain- and loss-of-function of the molecular mechanism and therapeutic application of miRNAs in cardiovascular disease, much more fruitful work about the molecular mechanism and therapeutic application of miRNAs in cardiovascular disease will emerge. We are confident that the diagnosis and therapeutics based on miRNA will play an even more important role in the field of gene therapy.
